# Decrease in COVID-19 adverse outcomes in adults during the Delta and Omicron SARS-CoV-2 waves, after vaccination in Mexico

**DOI:** 10.3389/fpubh.2022.1010256

**Published:** 2022-09-13

**Authors:** Lenin Domínguez-Ramírez, Itzel Solis-Tejeda, Jorge Ayon-Aguilar, Antonio Mayoral-Ortiz, Francisca Sosa-Jurado, Rosana Pelayo, Gerardo Santos-López, Paulina Cortes-Hernandez

**Affiliations:** ^1^Data Visualization Lab, Department of Chemical and Biological Sciences, School of Sciences, Universidad de las Américas Puebla, Puebla, Mexico; ^2^Population Health and Metadynamics Lab, Centro de Investigacion Biomedica de Oriente, Instituto Mexicano del Seguro Social, Puebla, Mexico; ^3^Coordinación Auxiliar Médica de Investigación en Salud, Jefatura de Prestaciones Médicas, Órgano de Operación Administrativa Desconcentrada Puebla, Instituto Mexicano del Seguro Social, Puebla, Mexico; ^4^Coordinación de Información y Análisis Estratégicos, Jefatura de Prestaciones Médicas, Órgano de Operación Administrativa Desconcentrada, Puebla, Instituto Mexicano del Seguro Social, Puebla, Mexico; ^5^Virology Lab, Centro de Investigacion Biomedica de Oriente, Instituto Mexicano del Seguro Social, Puebla, Mexico; ^6^Oncoimmunology and Cytomics Lab, Centro de Investigacion Biomedica de Oriente, Instituto Mexicano del Seguro Social, Puebla, Mexico

**Keywords:** COVID-19 in children, COVID-19 in pregnancy, COVID-19 vaccination, Omicron sub-lineages, SARS-CoV-2 Delta VOC, excess mortality, COVID-19 epidemic in Mexico

## Abstract

Mexico, one of the countries severely affected by COVID-19, accumulated more than 5. 1 all-cause excess deaths/1,000 inhabitants and 2.5 COVID-19 confirmed deaths/1,000 inhabitants, in 2 years. In this scenario of high SARS-CoV-2 circulation, we analyzed the effectiveness of the country's vaccination strategy that used 7 different vaccines from around the world, and focused on vaccinating the oldest population first. We analyzed the national dataset published by Mexican health authorities, as a retrospective cohort, separating cases, hospitalizations, deaths and excess deaths by wave and age group. We explored if the vaccination strategy was effective to limit severe COVID-19 during the active outbreaks caused by Delta and Omicron variants. Vaccination of the eldest third of the population reduced COVID-19 hospitalizations, deaths and excess deaths by 46–55% in the third wave driven by Delta SARS-CoV-2. These adverse outcomes dropped 74–85% by the fourth wave driven by Omicron, when all adults had access to vaccines. Vaccine access for the pregnant resulted in 85–90% decrease in COVID-19 fatalities in pregnant individuals and 80% decrease in infants 0 years old by the Omicron wave. In contrast, in the rest of the pediatric population that did not access vaccination before the period analyzed, COVID-19 hospitalizations increased >40% during the Delta and Omicron waves. Our analysis suggests that the vaccination strategy in Mexico has been successful to limit population mortality and decrease severe COVID-19, but children in Mexico still need access to SARS-CoV-2 vaccines to limit severe COVID-19, in particular those 1–4 years old.

## Introduction

SARS-CoV-2 was first detected in Mexico in February 2020 (1, 2). A large national epidemic ensued, with over 325,000 deaths confirmed from COVID-19 (2) and 662,000 all-cause excess deaths (3) in 2 years. In 2020–2021, Mexico ranked in the top-5 countries in excess deaths (4, 5) and in the top-30 in COVID-19 mortality (6), with 5.1 all-cause excess deaths and 2.5 COVID-19 confirmed deaths in every 1,000 inhabitants, similar to other severely hit countries like the USA, Brazil, Peru or Russia (1, 4–6).

Mexico's response to the pandemic in 2020 focused on organizing public medical attention for severe COVID-19 (1, 7), and less on infection detection or containment; while in 2021–2022 the focus was on anti-SARS-CoV-2 vaccination (8, 9). The country has endured five well-defined incidence waves, peaking approximately every 6 months. The first two waves happened before vaccination; the third wave presented in parallel to growing vaccination and to the colonization of the Delta SARS-CoV-2 variant (B.1.617.2), while the fourth wave begun with >70% of adults fully vaccinated (primary series), and correlated with the spread of Omicron subvariants BA.1 and BA.2 (10, 11). A fifth wave is developing at the time of writing, with presence of subvariants BA.4 and BA.5 (10).

Mexico began anti-SARS-CoV-2 vaccination on December, 2020, for healthcare workers and on February, 2021 for the adult population in age-groups from older to younger (8, 9). Up to April 2021, vaccination was only open to adults 60+ (11.5% of the population) and on May, June, July and August 2021, it opened for age groups 50–59, 40–49, 30–39 and 18–29 yo, which represent 10, 13, 15, and 20% of the population, respectively. Vaccination opened in May 2021 for pregnant people, in October 2021 for children 12–17 yo with severe comorbidities, in December 2021 for all children 15–17 yo and in May 2022 for those 12–14 yo; while children 5–11 yo (17% of population) will be vaccinated during July-September 2022, and children under 5 yo (8% of population) remain ineligible.

Mexico has relied on 7 COVID-19 vaccines from multiple developers: ChAdOx1 (AZD1222) from Oxford/Astra Zeneca (43.8%), BNT162b2 from Pfizer/BioNTech (25.5%), CoronaVac from Sinovac (9.9%), Gam-COVID-Vac/Sputnik V from Gamaleya Research Institute (9.9%), Ad5-nCoV from CanSino Biologics (7.0%), mRNA-1273 from Moderna (3.1%) and Ad26.COV2.S from J&J/Janssen (0.7%) (% of the initial 200 million doses received in the country until April 2022) (12). Full vaccination consisted of a single dose J&J/Janssen or CanSino, or of two doses of the rest of the vaccines, administered 4–6 weeks apart for Pfizer and Coronavac, and 9–12 weeks apart for Astra Zeneca and Sputnik V. Vaccines have been allocated as they arrive, without a strategy to serve age groups with a specific vaccine subtype, except for children <18 yo, all of whom have received BNT162b2 (Pfizer/BioNTech). Teachers and school personnel were offered immunization ahead of their age-group, in April-May 2021 with the single dose CanSino vaccine, followed by an mRNA-1273 Moderna booster 8 months later (offered on January 2022). All immunizations have been voluntary and offered at no cost to the population and no vaccine mandates are in place. Booster doses became available for adults, 5 months or more after their primary vaccination, with ChAdOx1 (Oxford/AstraZeneca, >85% of boosters administered), Sputnik V (Gamaleya) or Cansino; starting on December 2021 for those 60+, as the Omicron variant was identified in the country, and subsequently opening in January, February and March 2022, for 50–59 yo, 30–49 yo and 18+, respectively. Likely, most boosters have been heterologous, but we found no reports that specify this.

Mexico represents an interesting middle-income scenario to explore if a multi-vaccine strategy focused on immunizing and boosting the older population first, was effective to limit COVID-19 mortality during the active outbreaks caused by the Delta and Omicron variants. Here we analyzed the complete national data for the first four COVID-19 waves and correlated with the progress of anti-SARS-CoV-2 vaccination to describe how vaccination changed events per age group (cases, hospitalizations, deaths and excess deaths) during the COVID-19 epidemic in Mexico.

## Methods

### Ethics

The protocol describing this work was approved by IMSS ethics committee (registration IMSS-R-2021-2106-001). In all datasets patient identity was absent.

### Information sources

#### National COVID-19 dataset

COVID-19 cases, hospitalizations and deaths were obtained from the open-access Mexican dataset updated regularly by health authorities, available at (13). The dataset includes all symptomatic COVID-19 cases, their characteristics, and outcomes, since outbreak start. It is fed nationwide by all hospital centers, public and private, and it is the source of the official COVID-19 data provided by Mexican health authorities for international surveillance. Variable descriptors for the dataset are in an auxiliary file provided by the health authority (14) and we used those definitions without modification.

We analyzed the first four COVID-19 waves, defined as cases that started symptoms up to April 30, 2022, since an increase in COVID-19 cases (2) and test positivity (15) started around May 1, 2022, marking the beginning of a fifth COVID-19 wave. Individuals with symptoms, without a SARS-CoV-2 test result, were excluded. Individuals with symptoms that tested negative to SARS-CoV-2, were not included as COVID-19 cases but were used in test positivity calculations and as a reference negative population in **Figure 4D**. Individuals may appear more than once in the dataset if they had COVID-19 symptoms more than once during the 27 months of study. Reinfections or vaccination status of individuals are not marked in the national data set.

Final collection of the national dataset, was conducted on July 10, 2022 (16,922,254 entries), thus patient outcomes are known until that date (10 weeks after the last date of symptom onset). With the criteria described, we included 5,757,714 COVID-19 cases (52.4% females), from Feb 2020 to April 2022, that were confirmed by SARS-CoV-2 RT-PCR (35.1%), antigen test (57.0%), both (2.1%), or clinical/epidemiological evidence (5.8%). These cases generated 681,899 hospitalizations and resulted in 325,433 deaths (38.5% females). In the same time interval, 9,442,983 individuals (54.4% females), had symptoms but tested negative.

**Figure 1:**
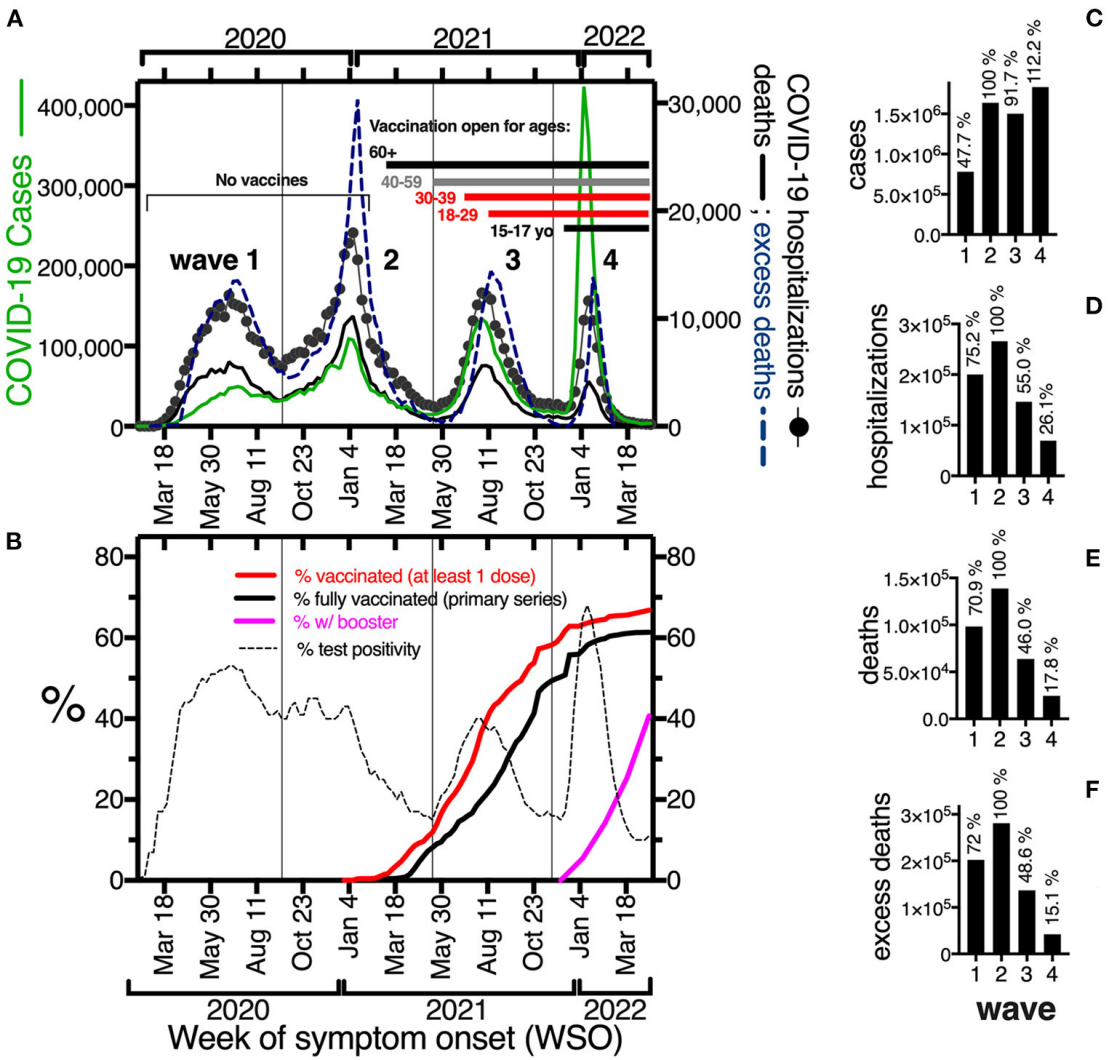
**(A)** Epidemic curve of COVID-19 cases (green line, left y-axis), COVID-19 hospitalizations (line with black circles) and deaths (bold black line); and all cause-excess deaths (blue discontinuous line) in the right y-axis, during the first 27 months of epidemic (Feb 2020–April 2022) in Mexico, per week of symptom onset, including all ages. **(B)** Percent test positivity for SARS-CoV-2 (discontinuous line), and percent population vaccinated with at least one dose (red line), a complete primary series (bold black line) or a complete primary series plus a booster (pink line), in the same period as in **(A)**. Vertical lines separate waves. **(C–F)** Accumulated COVID-19 cases, hospitalizations, deaths and all cause-excess deaths per wave. The percentages on top of bars were calculated using the second wave as 100%.

#### Excess mortality

All cause excess deaths were obtained from the official report by Mexican health authorities published at (3). Final data collection was on June 27, 2022 and included the dataset updated by health authorities on May 29, 2022 that covered data until epidemiological week 13 of 2022. Excess mortality was calculated by week as the difference between total deaths and expected deaths. For expected deaths, we used the 90 percentile and a model proposed by health authorities, both reported at (3). Both calculations were similar and included in graphs.

#### Analysis

The national dataset was analyzed as a retrospective cohort. COVID-19 cases, hospitalizations and deaths were organized by date of symptom onset, computed per week and separated into age groups 0–19, 20–39, 40–59 and 60+ yo in R (16)/R Studio (17) with script “DatesExtractionWorking.R” that can be found at (18). No modification was operated on the database other than filtering. A computer with at least 32 Gb of RAM is required for this step. The script above is dependent on R libraries dplyr (19) and tidyverse (20). Briefly, data was first separated according to diagnosis, then by date of death, or its absence. Then, groups were filtered by age and, counts per group were added by week within the periods selected, based on week of symptom onset.

Peaks in epidemic curves were detected automatically with software Magicplot, by fitting to gaussian and verified manually (see Supplementary material for details).

Wave dates were: (1) Feb-16-2020–Sept-19-2020 (epidemiological weeks 7–37 of 2020); (2) Sept-20-2020–May-15-2021 (epi weeks 38 of 2020 to 19 of 2021); (3) May-16-2021–Nov-20-2021 (epi weeks 20-46 of 2021); (4) Nov-21-2021–Apr-30-2022 (epi weeks 47 of 2021 to 17 of 2022). Dates of start of each wave were determined by finding the point of inflection, that is the week when numbers of cases and hospitalizations increased with respect to the previous week, after 10+ weeks of descent.

The following were calculated per week: percent of cases that were hospitalized; measured case fatality rate (CFR) which was the % of identified cases that died; CFR of the hospitalized; test positivity, which was the % of positive tests from the total conducted and was verified against data per week published by health authorities (15). Vaccination coverage was calculated as (number of people vaccinated^*^100)/(population). Vaccines applied and people vaccinated with one or more doses, were as reported by health authorities and verified in the COVID-19 OWID data set (21), per date. Population estimates per age group to calculate rates, were obtained from populationpyramid.net (22) for 2021 (total Mexican population 130,262,220) and verified against reports by the Mexican government (23).

## Results

### Four COVID-19 waves in two years of epidemic in Mexico: National data

In 27 months (Feb 2020–April 2022), Mexico experienced four COVID-19 waves, with cases, hospitalizations, deaths and excess deaths peaking every 6 months, in summer and winter (Figure 1A). Up to April 30, 2022, Mexico identified officially almost 5.8 million cumulative COVID-19 cases, but high seroprevalence (24, 25), high test-positivity (Figure 1B) and high case-fatality (**Figure 4A**) suggest cases were under detected. Each wave happened under unique conditions, including the predominance of a particular SARS-CoV-2 variant, different mobility restrictions (more intense in the first surges and decreasing gradually) and likely different case-detection levels, with the lowest detection in the first wave as fewer tests were available. More important, no vaccination was available in the first two waves, and vaccination coverage grew during waves 3 and 4 (Figure 1B). Fifty million cumulative vaccine doses were administered by July 2021, 100 million by September 2021 and 200 million by April 30, 2022, when 90% of adults 18+ had at least one dose of a SARS-CoV-2 vaccine, while 59% had complete primary vaccination plus a booster. Vaccination coverage in children lagged, and around 40% of children 12–17 yo received at least one vaccine dose by April 30, 2022, while individuals 5–11 became eligible, only after the period analyzed.

To evaluate the magnitude of the SARS-CoV-2 surges and discuss the effect of vaccination, we compared the counts in each wave against the second wave, which had the most adverse outcomes (Figures 1C–F). Despite different durations (34, 27 and 23 weeks, respectively Supplementary Table 1), the last 3 waves had a similar number of detected cases, around 1.6 million; while the first wave had half as many cases (Figure 1C), related in part to less testing. In contrast, hospitalizations, deaths and excess deaths declined in waves 3 and 4 (Figures 1D–F), after vaccination.

### Age-group analysis of the COVID-19 waves and the population effect of vaccination

**Figure 2:**
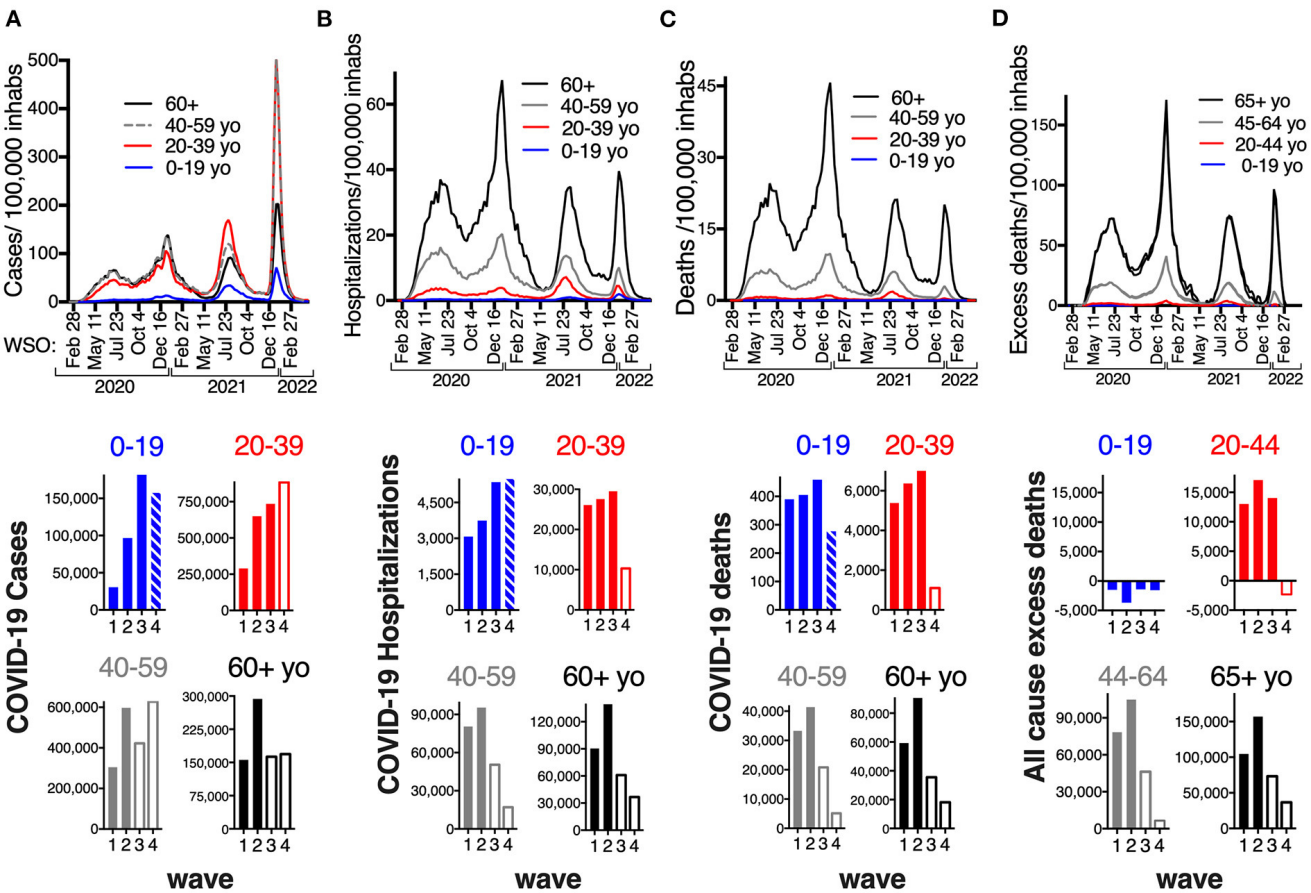
Rates per 100,000 inhabitants of COVID-19 cases **(A)**, hospitalizations **(B)**, deaths **(C)** and all-cause excess deaths **(D)**, per epidemiological week of symptom onset (WSO), per age group (0–19 yo blue; 20–39 yo red; 40–59 yo gray; 60+ black), in the first 27 months of epidemic in Mexico (Feb 2020–April 2022). Bottom: Accumulated cases hospitalizations, deaths and all cause excess deaths, per wave per age group. Bars are empty when the age-group had access to COVID-19 vaccination. The last bar in age group 0–19 represents that 15–19 yo had access to vaccination but not the rest of the children, 15–17 yo accessed first doses in December 2021; while 18–19 yo were vaccinated with the adults beginning on late August 2021, thus had the chance to complete a primary series before the fourth (Omicron) wave.

A clearer picture of the effect of vaccination emerges when separating the analysis per age group (Figure 2). In the first two waves, older age groups had a higher case rate, while in the third wave, adult age groups inverted their positions, with lower case rate in those that accessed vaccination first (Figure 2A). The older population in Mexico (60+) had the most adverse COVID-19 outcomes in all waves (Figures 2B,C), accounting for 48% COVID-19 hospitalizations and 62.5% COVID-19 deaths in the period analyzed (Supplementary Table 1). This age group was the only to access a full-vaccination primary series before the third wave (Delta) (estimated coverage 76% of the age group by the end of May 2021); while 40–59 yo were offered vaccination as the third wave developed (May-August 2021). Vaccination of just those age groups (the eldest 34% of the population) importantly reduced severe COVID-19, halving hospitalizations, deaths and excess deaths in the third wave relative to the second (Figures 1D–F), despite similar numbers of identified cases (Figure 1B).

By December 2021, when the Omicron variant colonized the country, all adult groups in Mexico had accessed vaccines and those 60+ had accessed a booster or third dose, 5–8 months after their primary series. Even with vaccine coverage >70% in adult groups, and booster coverage 50% for the 60+, a large fourth wave developed early in 2022, driven by fast Omicron (BA.1, BA.2) transmission, but with fewer adverse outcomes. Hospitalizations, deaths and excess deaths were much lower than in previous waves (26, 17, and 15% of the second wave, respectively, Figures 1D–F).

The reduction in severe outcomes in waves 3 and 4 came from the age groups that accessed vaccination (Figures 2B–D, bottom; Supplementary Table 1). In wave 3, the adult groups that had accessed vaccination (40–59 yo and 60+), had less than half of the hospitalizations and deaths relative to wave 2. In contrast, adults under 40 yo accessed first vaccine doses in the second half of the third wave, so they faced wave 3 with little to no protection from vaccines and actually had more COVID-19 hospitalizations (107%) and deaths (110%), and almost as many excess deaths (82%) as in the second wave. This group (20–39 yo) only decreased their hospitalizations and fatalities in the fourth wave, after their complete vaccination, and their excess deaths ceased (Figures 2B–D bottom).

**Figure 3:**
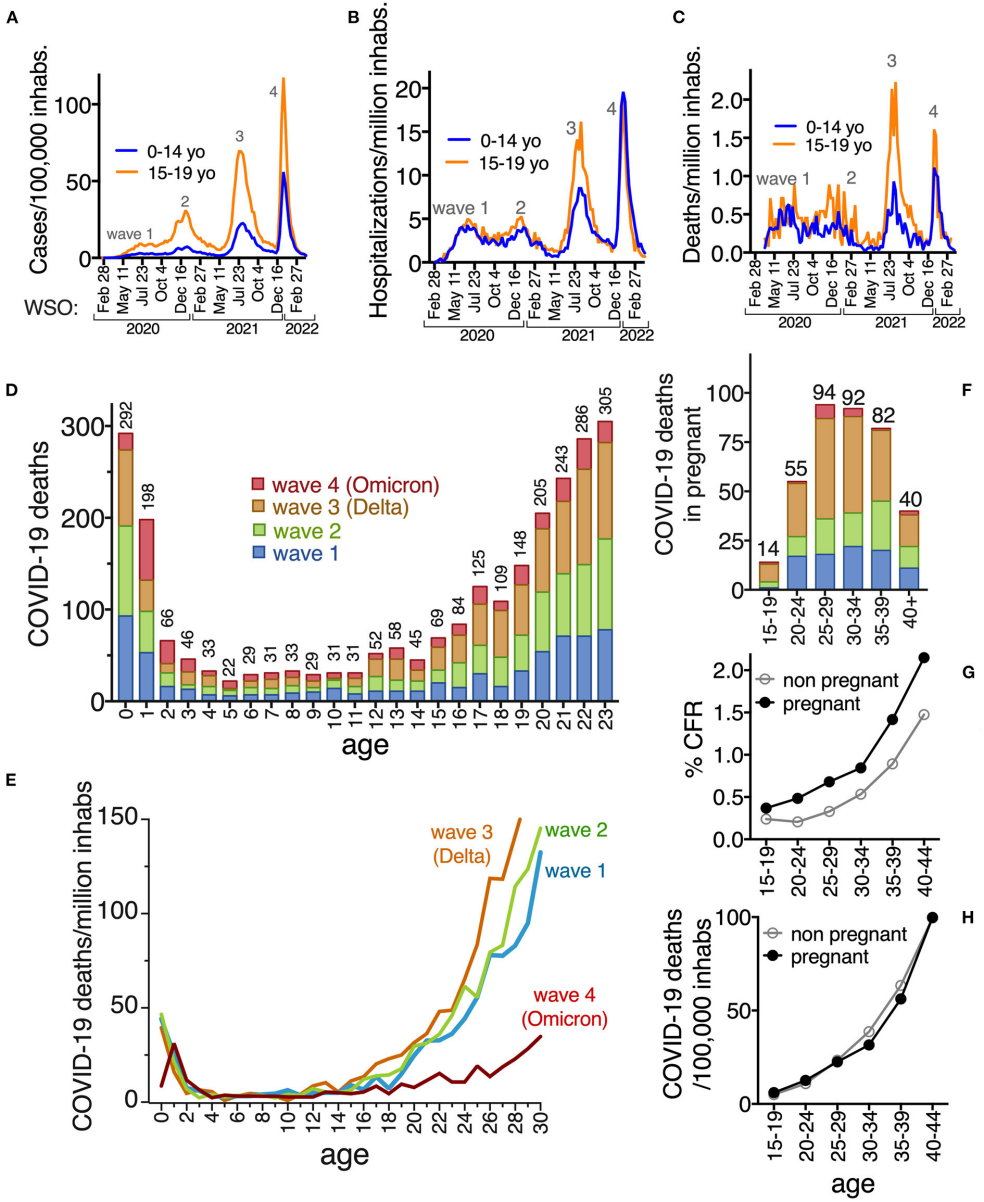
Rates of COVID-19 cases per 100,000 inhabitants **(A)**, hospitalizations **(B)** and deaths **(C)** per million inhabitants, in children 0–14 yo (blue line) that were ineligible for COVID-19 vaccinations in the period analyzed, compared to those 15–19 yo (orange line) that had access to vaccines before or during the fourth wave. **(D)** COVID-19 deaths identified in children, teens and young adults, per age, per wave and **(E)** death rates per million inhabitants comparing waves. In **(D)** the graph shows up to age 23 to appreciate that the cumulative number of deaths in infants is similar to those in 22 and 23 yo. In **(E)** the decrease in deaths in infants 0 yo during the fourth wave is obvious, and more ages were graphed, to show the decrease in fatalities after age 18 during the fourth wave. **(F)** COVID-19 deaths identified in pregnant people, by age-group and wave; **(G)** % Case fatality rate (CFR) and **(H)** deaths/100,000 inhabs in pregnant and non-pregnant. The same color code is used per wave in **(D–F)**. Numbers on top of bars are the total of deaths confirmed as COVID-19, reported in the national dataset at each age, up to April 2022 (including all four waves analyzed).

Likewise, most children under 18 yo did not access full vaccination in Mexico until 2022, so the age group 0–19 also had more hospitalizations (143%) and deaths (113%) in the third wave (Delta) than in the second, and this pattern persisted for the fourth wave, which had 146% hospitalizations relative to the second (Figure 2B, bottom, Supplementary Table 1). First dose vaccination opened for 15–17 yo during the fourth wave, while younger children remained ineligible, so most children faced the Omicron wave without complete vaccination. However, less COVID-19 fatalities happened in 0–19 yo during the Omicron wave, than in previous waves (68.4% of the second wave, Figure 2C, bottom), related to: (1) less infant (0 yo) deaths with Omicron (Figures 3D,E); (2) less deaths in 15-19 yo (Figure 3C), and in particular in 18–19 yo (Figure 3D) that accessed complete vaccination as adults, shortly before the Omicron wave; (3) shorter wave duration. Pediatric age groups 0–14 and 15–19 (with and without vaccine access), had similar low hospitalization and death rates from COVID-19 in the first two waves, which increased in waves 3 and 4 (Figures 3B,C). The increase was larger for 15–19 yo in the third wave, but in the fourth wave, hospitalizations and fatalities were again similar between these two groups, perhaps related to the access of 15–19 yo to vaccinations shortly before the fourth wave.

In the period analyzed, there were almost half a million detected cases in children 0–19 yo, that resulted in 17,644 hospitalizations and 1,531 COVID-19 deaths (45.2% females) Of these deaths 635 (41.5%) were 0–4 yo and 748 (48.9%) 5–18 yo. Ages 0 and 1 yo accumulated the most deaths (Figure 3D) and most of the children that died from COVID were previously healthy (no comorbidities reported in 62.5% of those 0–4 yo and in 53.2% of 0–19 yo that died). COVID-19 deaths distributed similarly through pediatric ages during the first three waves, whereas in wave 4 (Omicron), there was a decrease in deaths around age 15, more prominent in ages 18+, consistent with vaccination access (Figure 3E).

Additionally, in wave 4 there was a large decrease in deaths in infants 0 yo, but not in those 1 and 2 yo who had more deaths in the fourth than in previous waves (Figures 3D,E). The decrease in infant (0 yo) fatalities could correspond to the vaccination of pregnant people that started in May 2021, and that correlated with an important decrease in COVID-19 deaths in the pregnant by the fourth wave (Figure 3F). In the period analyzed, there were 47,671 COVID-19 cases identified in pregnant people, that resulted in 7,366 hospitalizations and 377 COVID-19 deaths (CFR 0.79%; 65.5% of deaths without previous comorbidity), 50% of them during wave 3, but decreasing for wave 4, which had only 16 deaths (4.2% of total). The CFR for the pregnant was higher than for non-pregnant females of the same age (Figure 3G), resulting in a Relative Risk of death = 1.2 for being pregnant (95% CI 1.08–1.32), higher at ages 20–30 yo (Supplementary Table 2). Mortality rates in the pregnant were similar than in non-pregnant females, ranging from 6 to 100 deaths/100,000 pregnancies, and highly dependent on maternal age (Figure 3H; Supplementary Table 2).

**Figure 4:**
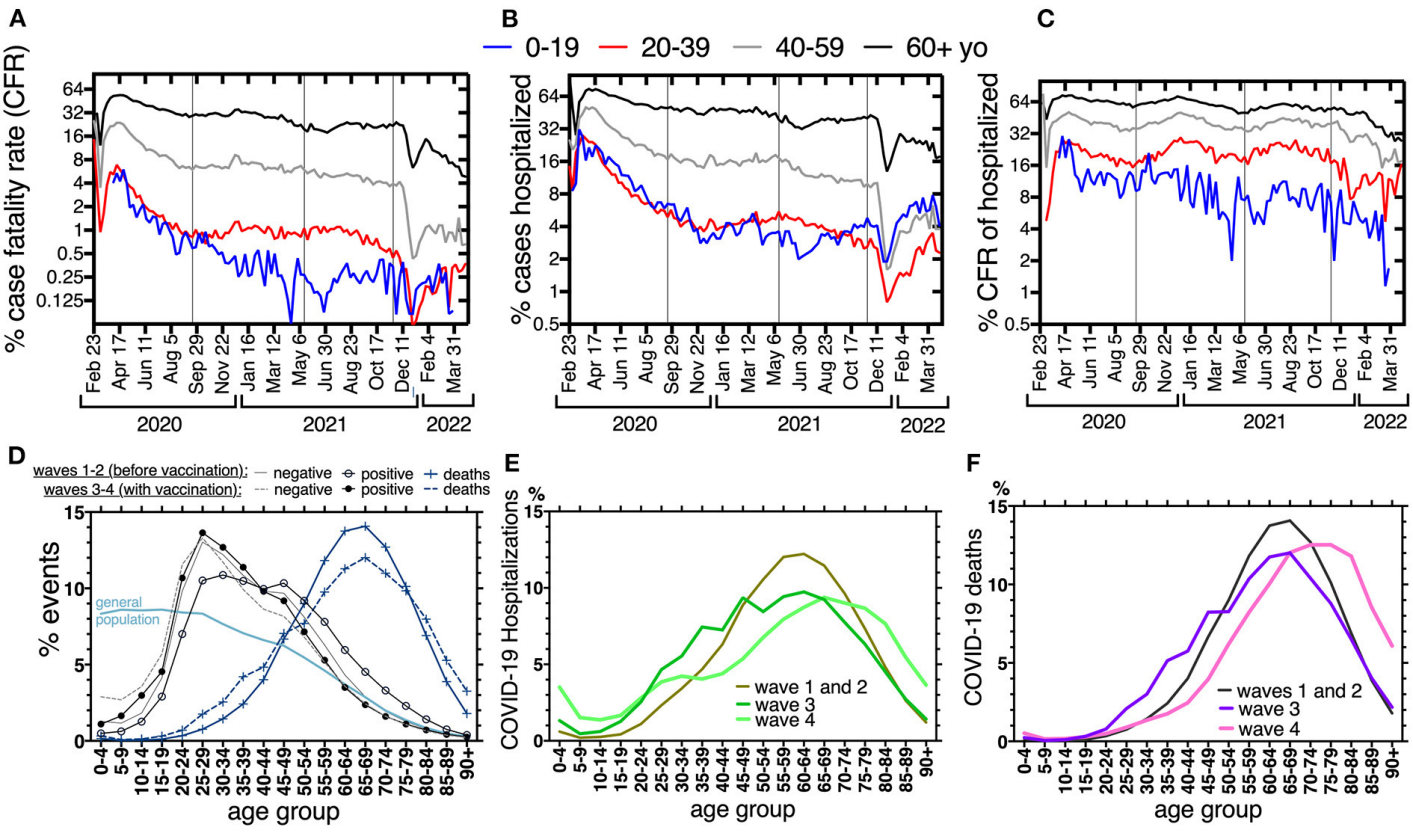
**(A)** Percent case fatality rate (CFR), **(B)** percent cases that were hospitalized and **(C)** percent case fatality rate of those hospitalized, per week of symptom onset, across the first four waves (Feb-2020–Apr-2022), separated by age group: 0–19 yo blue lines, 20–39 yo red lines, 40–59 yo gray lines, 60+ black lines. In **(A–C)** y-axes are log 2, to favor curve display, and horizontal lines separate the waves, like in Figures 1A,B. **(D–F)** are the percent events separated into 5-year age groups, to appreciate age distribution across the population and waves. Waves 1 and 2, before vaccines, had a similar distribution and thus are displayed together. In **(D)**, waves 3 and 4 (when vaccination of adults happened) are displayed together. In **(D)**, events are: individuals with symptoms that tested negative or positive to SARS-CoV-2 and those that died from confirmed COVID-19. The pale blue line represents the age-distribution of the population pyramid in Mexico 2021 (“general population”), for reference. In **(E,F)** only COVID-19 hospitalizations and deaths are displayed, respectively. Waves 3 and 4 are separated to appreciate the differences in distribution as vaccination progressed.

Despite being the last population group to access vaccines, no excess mortality has been identified in children in Mexico (Figure 2D), and their population rates of hospitalization and death from COVID-19 remain lower than in adult groups (Figures 2B,C), consistent with the severity gradient that has been identified for COVID-19 with age. However, children were the only age group that did not show a drop in case fatality rate (CFR) (Figure 4A) or in the % of cases within the age group requiring hospitalization, during the Omicron wave (Figure 4B). In fact, the % of pediatric cases hospitalized grew with Omicron, surpassing the % of cases that required hospitalization in adult age-groups 20–29 and 40–59 yo, for the first time in the epidemic (Figure 4B).

The age distribution of the national COVID-19 events is driven by the population pyramid and by the age severity gradient of the disease, thus it changed only slightly before and after vaccination (Figure 4D). The curve of individuals that had symptoms but tested negative, showed no change with vaccination, while the peak of cases positive to SARS-CoV-2 switched to younger ages after vaccination and overlapped with that of individuals that tested negative (Figure 4D). After vaccination, the frequency of hospitalizations and deaths decreased at ages 40–80 and increased slightly at younger ages, again correlating with latter vaccination access of the younger half of the population. During the fourth wave, when all adults had accessed vaccination, the age distribution of hospitalizations and deaths shifted toward older ages (Figures 4E,F). However, hospitalizations in the fourth wave retained a component from ages below 35 yo, perhaps related to lower coverage of primary vaccination or boosters in the younger adults. An important increase in the frequency of hospitalizations in 0–4 yo is also seen in the fourth wave (Figure 4E). The curve of deaths in the fourth wave does not have those young-age components (Figure 4F), suggesting that most of the hospitalizations in the young result in improvement. Accordingly, the CFR of young hospitalized cases (0–19 and 20–39), remained lower than for older groups, across the four waves (Figure 4C).

## Discussion

Mexico was one of the countries most affected by COVID-19 in 2020–2021 (1, 4, 5), with substantial fatalities and excess mortality. In 2020 and 2021, heart disease and COVID-19 were the first causes of death in Mexico with a narrow margin between them (26). Vaccination changed this trend and a clear decrease in COVID-19 deaths and hospitalizations is evident starting on the third wave driven by Delta SARS-CoV-2 in mid-2021, when vaccine access for the eldest third of the population resulted in a 50% decrease in COVID-19 deaths and hospitalizations. Further decrease was observed in 2022 during the Omicron wave when all adults had accessed vaccines, with 82% decrease in COVID-19 deaths and 85% in excess mortality compared to the second wave, despite a similar number of cases detected.

As of July 2022, >90% of the 18+, >60% of 12–17 yo and 25% of those 5 to 11 yo have received at least one dose of COVID-19 vaccines. Additionally, serology in the Mexican population suggested extensive SARS-CoV-2 circulation since 2020, in all age groups (24, 25). Thus, the population has been immunized both by vaccination and by infection/reinfection. As in other parts of the world, this immunization hasn't been enough yet to prevent further waves of SARS-CoV-2, as new variants arise. Yet a clear pattern of decreased adverse outcomes is noticeable as each age group accessed vaccination, suggesting that vaccination had a strong protective effect against hospitalization and death on the population, on top of the immunity by natural infection.

As a middle-income country, vaccination of the Mexican population has been a challenge amid international competition for vaccines and 7 different vaccines have been used, as described in the introduction. Our analysis suggests that this strategy to use vaccines from different developers, as available, has been successful to limit mortality. All of these vaccines were based on the ancestral SARS-CoV-2 but show good population effectiveness to decrease severe forms of the disease from all the variants so far, and despite the fact that only 60–68% of the vaccinated adults have received a booster, most with vaccines that do not use mRNA technology.

For 13 weeks starting on May 1, 2022, Mexico has been undergoing a fifth COVID-19 wave that seems to have reached its peak, adding almost one million more cases, but < 20,000 hospitalizations (43.95% in 60+) and < 2,000 deaths (76.6% in 60+), thus projecting as the least lethal wave so far, although complete data and appropriate time to discern patient outcomes is needed to analyze this accurately. The epidemiological behavior of the fifth wave suggests that the protective effect of immunization prevails.

Despite good vaccination coverage and access to boosters, the eldest individuals continue to accumulate the most adverse outcomes and are at greater risk of adverse outcomes than younger population. Thus, the most labile individuals should be alerted to limit their community exposure during high SARS-CoV-2 circulation. A limitation of our analysis is that we cannot distinguish if the individuals that died from COVID-19 were vaccinated and further studies are needed to evaluate the real-world effectiveness of the vaccines used.

At the other end of the age spectrum, the pediatric population in Mexico (except for infants 0 yo) has not yet seen a clear decrease in COVID-19 adverse outcomes, likely because their vaccination has lagged. Pediatric COVID-19 hospitalizations in Mexico almost doubled from 2020 to 2021 from 4,895 to 7,905 and the trend hasn't changed yet for 2022 which in 7 months accumulates 7,779 COVID-19 hospitalizations. Mexico has 44.4 million inhabitants 0–19 yo and 1,531 COVID-19 deaths at these ages during the first four waves, plus 46 deaths in children 0–19 added so far in the fifth wave (18 of which were in 0–4 yo). This results in a cumulative death rate of 3.6/100,000 inhabs 0–19 yo, which is roughly double that of the USA, that has 1.7 deaths/100,000 inhabs 0–18 yo [1,325 COVID-19 deaths (27) and 78 million inhabs 0–18 (28)]. Death rate from COVID-19 is higher in population 0–4 yo, currently ineligible for vaccination in Mexico, which accumulates 6 deaths/100,000 inhabs in Mexico vs. 2.4/100,000 in the USA (27). These pediatric COVID-19 death rates are orders of magnitude lower than in adults in Mexico, who have accumulated 48.8, 338.2 and 1,363.7 deaths/100,000 inhabs 20–39, 40–59 and 60+, respectively (Supplementary Table 1). Yet, COVID-19 figures in the first ten pediatric death causes in Mexico, although at lower rates than causes like accidents, cancer, congenital malformation, and neurological disease. Analyses to discern if severe respiratory infections by other pathogens persisted during the COVID-19 epidemic are lacking in Mexico. Also, MIS-C (Multisystem Inflammatory Syndrome in Children) and long COVID-19 haven't been analyzed in the Mexican population and are not registered in the national COVID-19 data base, that reports only the result of acute infections. Our analysis further suggests that vaccination of pregnant people and young people of reproductive age, resulted in a decrease of 85–90% in deaths in the pregnant and 80% in infants 0 yo, by the fourth wave.

All in-person education closed in Mexico for 17 months from March/21/2020 to August/29/2021, and its impact on the epidemic hasn't been measured. Children had less hospitalizations and deaths in the first two waves, when schools were closed, but the increase in pediatric adverse outcomes in wave 3 started months before school re-openings, when the Delta wave grew nationally (Figures 3A–C), so it seemed to respond more to community circulation of the virus than to school re-openings.

Several reports suggest that initial Omicron subvariants were clinically less severe than previous variants (29–34). In Mexico, the progress in vaccination coverage could explain the decrease in severe outcomes during the Omicron wave, and the dataset analyzed doesn't contain individual information on vaccination to correctly explore Omicron severity. However, age groups 1–14 yo without access to complete vaccination before Omicron circulation, did not experience a decrease in COVID-19 deaths during the Omicron wave. In fact, 1 and 2 yo had 50% more fatalities than in previous waves arguing against Omicron mildness. As new waves of COVID-19 sweep the country, the unvaccinated population will be at risk of severe COVID-19, thus vaccine access for all the population is crucial to prevent hospitalizations and deaths, in particular in children.

## Data availability statement

The datasets presented in this study can be found in online repositories. The names of the repository/repositories and accession number(s) can be found below: https://www.gob.mx/salud/documentos/datos-abiertos-152127.

## Ethics statement

The studies involving human participants were reviewed and approved by Instituto Mexicano del Seguro Social 2106. Written informed consent from the participants' legal guardian/next of kin was not required to participate in this study in accordance with the national legislation and the institutional requirements.

## Author contributions

PC-H: conceptualization. PC-H, LD-R, JA-A, and AM-O: methodology. LD-R and IS-T: data curation. LD-R, PC-H, and IS-T: formal analysis. PC-H, GS-L, IS-T, JA-A, and FS-J: investigation. PC-H and LD-R: writing—original draft preparation. PC-H, LD-R, FS-J, RP, and GS-L: writing—review and editing. PC-H and LD-R: supervision. All authors have read and agreed to the published version of the manuscript.

## Funding

This work was funded by IMSS (Insituto Mexicano del Seguro Social, Coordinación de Investigación en Salud) covered publication costs through its program: Apoyo económico para la publicación de artículos científicos en revistas de alto impacto, 2022.

## Conflict of interest

The authors declare that the research was conducted in the absence of any commercial or financial relationships that could be construed as a potential conflict of interest.

## Publisher's note

All claims expressed in this article are solely those of the authors and do not necessarily represent those of their affiliated organizations, or those of the publisher, the editors and the reviewers. Any product that may be evaluated in this article, or claim that may be made by its manufacturer, is not guaranteed or endorsed by the publisher.
