# Supplementary material for: Decrease in COVID-19 adverse outcomes in adults during the Delta and Omicron SARS-CoV-2 waves, after vaccination in Mexico
Source: Front Public Health. 2022 Sep 13;10:1010256. doi: 10.3389/fpubh.2022.1010256 (PMC9513220; doi:10.3389/fpubh.2022.1010256)
Supplement: Supplementary file 1 [file Table_1.docx]

**Supplementary Table 1. COVID-19 cases, hospitalizations and deaths per wave and age group in Mexico from February 2020 to April 2022.**

|  | | | AGE GROUPS | | | |  |
| --- | --- | --- | --- | --- | --- | --- | --- |
|  | | | 0-19 yo | 20-39 yo | 40-59 yo | 60+ yo | Total,  ALL AGES |
| IDENTIFIED COVID-19 CASES (POSITIVE TO SARS-CoV-2) | | |  |  |  |  |  |
| First wave (31 weeks) | | n (% of the wave) | 30,633 (3.9) | 289,645 (37.1) | 304,589 (39.0) | 155,996 (20.0) | 780,863 (100) |
|  |  | n per week | 988.2 | 9,343.4 | 9,825.5 | 5,032.1 | 25,189.1 |
|  |  | % of second wave | 31.7 | 44.6 | 51.0 | 53.1 | 47.7 |
|  | | cases/1,000 inhabs | 0.69 | 7.07 | 10.21 | 10.38 | 5.99 |
| Second wave (34 weeks) | | n (% of the wave) | 96,756 (5.9) | 650,034 (39.7) | 596,987 (36.5) | 293,577 (17.9) | 1,637,354 (100) |
|  |  | n per week | 2,764.5 | 18,572.4 | 17,056.8 | 8,387.9 | 46,781.5 |
|  |  | % of second wave | 100 | 100 | 100 | 100 | 100 |
|  | | cases/1,000 inhabs | 2.18 | 15.86 | 20.01 | 19.53 | 12.57 |
| Third wave (27 weeks) | | n (% of the wave) | 181,770 (12.1) | 735,120 (49.0) | 422,207 (28.1) | 162,786 (10.8) | 1,501,883 (100) |
|  |  | n per week | 6,732.2 | 27,226.7 | 15,637.3 | 6,029.1 | 55,625.3 |
|  |  | % of second wave | 187.9 | 113.3 | 70.7 | 55.4 | 91.7 |
|  | | cases/1,000 inhabs | 4.09 | 17.93 | 14.15 | 10.83 | 11.53 |
| Fourth wave (23 weeks) | | n (% of the wave) | 157,399 (8.6) | 883,869 (48.1) | 627,385 (34.1) | 168,961 (9.2) | 1,837,614 (100) |
|  |  | n per week | 6,843.4 | 38,429.1 | 27,277.6 | 7,346.1 | 79,896.3 |
|  |  | % of second wave | 162.7 | 136.0 | 105.1 | 57.6 | 112.2 |
|  | | cases/1,000 inhabs | 3.54 | 21.56 | 21.03 | 11.24 | 14.11 |
| **Total, all four waves**  **(115 weeks)** | | **n (% of the wave)** | **466,558 (8.1)** | **2,558,668 (44.4)** | **1,951,168 (33.9)** | **781,320 (13.6)** | **5,757,714 (100)** |
|  |  | **n per week** | **4,057.0** | **22,249.3** | **16,966.7** | **6,794.1** | **50,067.1** |
|  | **cases/1,000 inhabs** | | **10.51** | **62.42** | **65.39** | **51.99** | **44.20** |
| COVID-19 HOSPITALIZATIONS | | |  |  |  |  |  |
| First wave (31 weeks) | | n (% of the wave) | 3,075 (1.5) | 26,095 (13.0) | 80,578 (40.3) | 90,366 (45.2) | 200,114 (100) |
|  |  | n per week | 99.2 | 841.8 | 2,599.3 | 2,915.0 | 6455.3 |
|  |  | % of second wave | 82.2 | 94.6 | 84.5 | 64.9 | 75.2 |
|  | | % cases hospitalized | 10.0 | 9.0 | 26.5 | 57.9 | 25.6 |
|  | | Hospitalizations/10,000 inhabs | 0.69 | 6.37 | 27.00 | 60.13 | 15.36 |
| Second wave (34 weeks) | | n (% of the wave) | 3,740 (1.4) | 27,572 (10.4) | 95,335 (35.8) | 139,305 (52.4) | 265,952 (100) |
|  |  | n per week | 106.9 | 787.8 | 2,723.9 | 3,980.1 | 7,598.6 |
|  |  | % of second wave | 100 | 100 | 100 | 100 | 100 |
|  | | % cases hospitalized | 3.9 | 4.2 | 16.0 | 47.5 | 16.2 |
|  | | Hospitalizations/10,000 inhabs | 0.84 | 6.73 | 31.95 | 92.69 | 20.42 |
| Third wave (27 semanas) | | n (% of the wave) | 5,350 (3.6) | 29,506 (20.2) | 50,455 (34.5) | 60,985 (41.7) | 146,296 (100) |
|  |  | n per week | 198.1 | 1,092.8 | 1,868.7 | 2,258.7 | 5,418.4 |
|  |  | % of second wave | 143.0 | 107.0 | 52.9 | 43.8 | 55.0 |
|  | | % cases hospitalized | 2.9 | 4.0 | 12.0 | 37.5 | 9.7 |
|  | | Hospitalizations/10,000 inhabs | 1.20 | 7.20 | 16.91 | 40.58 | 11.23 |
| Fourth wave (23 weeks) | | n (% of the wave) | 5,479 (7.9) | 10,307 (14.8) | 17,021 (24.5) | 36,730 (52.8) | 69,537 (100) |
|  |  | n per week | 238.2 | 448.1 | 740.0 | 1,597.0 | 3,023.3 |
|  |  | % of second wave | 146.5 | 37.4 | 17.9 | 26.4 | 26.1 |
|  | | % cases hospitalized | 3.4 | 1.2 | 2.7 | 21.2 | 3.7 |
|  | | Hospitalizations/10,000 inhabs | 1.23 | 2.51 | 5.70 | 24.44 | 5.34 |
| **Total, four waves**  **(115 weeks)** | | **n (% of the wave)** | **17,644 (2.6)** | **93,480 (13.7)** | **243,389 (35.7)** | **327,386 (48.0)** | **681,899 (100)** |
|  |  | **per week** | **153.4** | **812.9** | **2,116.4** | **2,846.8** | **5,929.6** |
|  |  | **% cases hospitalized** | **3.8** | **3.7** | **12.5** | **41.9** | **11.8** |
|  |  | **Hospitalizations/10,000 inhabs** | **3.97** | **22.81** | **81.57** | **217.84** | **52.35** |
| DEATHS CONFIRMED AS COVID-19 | | |  |  |  |  |  |
| First wave (31 weeks) | | n (% of the wave) | 390 (0.4) | 5,385 (5.5) | 33,278 (33.8) | 59,238 (60.3) | 98,291 (100) |
|  |  | n per week | 12.6 | 173.7 | 1,073.5 | 1,910.9 | 3,170.7 |
|  |  | % of second wave | 96.3 | 84.5 | 80.4 | 65.4 | 70.9 |
|  | | % CFR | 1.3 | 1.9 | 10.9 | 38.0 | 12.6 |
|  | | deaths/100,000 inhabs | 0.88 | 13.14 | 111.53 | 394.17 | 75.46 |
| Second wave (34 weeks) | | n (% of the wave) | 405 (0.3) | 6,370 (4.6) | 41,370 (29.8) | 90,550 (65.3) | 138,695 (100) |
|  |  | n per week | 11.6 | 182.0 | 1,182 | 2,587.1 | 3,962.7 |
|  |  | % of second wave | 100 | 100 | 100 | 100 | 100 |
|  | | % CFR | 0.4 | 1.0 | 6.9 | 30.8 | 8.5 |
|  | | deaths/100,000 inhabs | 0.91 | 15.54 | 138.64 | 602.51 | 106.47 |
| Third wave (27 semanas) | | n (% of the wave) | 459 (0.7) | 7,027 (11.0) | 20,800 (32.6) | 35,505 (55.7) | 63,791 (100) |
|  |  | n per week | 17.0 | 260.3 | 770.4 | 1,315.0 | 2,362.7 |
|  |  | % of second wave | 113.1 | 109.9 | 50.2 | 39.1 | 45.9 |
|  | | % CFR | 0.3 | 1.0 | 4.9 | 21.8 | 4.2 |
|  | | deaths/100,000 inhabs | 1.03 | 17.14 | 69.71 | 236.25 | 48.97 |
| Fourth wave (23 weeks) | | n (% of the wave) | 277 (1.1) | 1,102 (4.5) | 5,133 (20.8) | 18,144 (73.6) | 24,656 (100) |
|  |  | n per week | 12.0 | 47.9 | 223.2 | 788.9 | 1,072.0 |
|  |  | % of second wave | 68.4 | 17.3 | 12.4 | 20.0 | 17.8 |
|  | | % CFR | 0.2 | 0.1 | 0.8 | 10.7 | 1.3 |
|  | | deaths/100,000 inhabs | 0.62 | 2.69 | 17.20 | 120.73 | 18.93 |
| **Total, four waves**  **(115 weeks)** | | **n (% of the wave)** | **1,531 (0.5)** | **19,884 (6.1)** | **100,581 (30.9)** | **203,437 (62.5)** | **325,433 (100)** |
|  |  | **n per week** | **13.3** | **172.9** | **874.6** | **1,769.9** | **2,829.85** |
|  |  | **% CFR** | **0.3** | **0.8** | **5.2** | **26.0** | **5.7** |
|  |  | **deaths/100,000 inhabs** | **3.45** | **48.51** | **337.08** | **1,353.66** | **249.83** |

Wave definitions were: 1) Feb-16-2020 to Sept-19-2020 (epidemiological weeks 7-37 of 2020); 2) Sept-20-2020 to May-15-2021 (epi weeks 38 of 2020 to 19 of 2021); 3) May-16-2021 to Nov-20-2021 (epi weeks 20-46 of 2021); 4) Nov-21-2021 to Apr-30-2022 (epi weeks 47 of 2021 to 17 of 2022).

Cases were confirmed by SARS-CoV-2 RT-PCR (35.1%), antigen test (57.0%), both (2.1%), or clinical/epidemiological evidence (5.8%)

CFR, Case fatality Rate.

Population estimates for rate calculations were: 0-19 yo: 44,403,837; 20-39 yo: 40,990,830; 40-59 yo: 29,838,834 and 60+:15,028,719. Total population: 130,262,220 according to (22).
