# Supplementary material for: Decrease in COVID-19 adverse outcomes in adults during the Delta and Omicron SARS-CoV-2 waves, after vaccination in Mexico
Source: Front Public Health. 2022 Sep 13;10:1010256. doi: 10.3389/fpubh.2022.1010256 (PMC9513220; doi:10.3389/fpubh.2022.1010256)
Supplement: Supplementary file 2 [file Table_2.docx]

**Supplementary Table 2.** COVID-19 cases, hospitalizations and deaths in pregnant and non-pregnant females in Mexico from February 2020 to April 2022.

|  | Non-Pregnant | | | | | | Pregnant | | | | | | | RR of death  [95% CI] ^c^ |
| --- | --- | --- | --- | --- | --- | --- | --- | --- | --- | --- | --- | --- | --- | --- |
| Age | Cases | Hospitalizations | | Deaths | | Deaths/  100,000 inhabs | Cases | Hospitalizations | | Deaths | | | Deaths/  100,000 live births ^b^ |  |
|  | N | N | % cases hospitalized | N | CFR |  | N | N | % cases hospitalized | | N | CFR |  |  |
| 15-19 yo | 111,016 | 2,254 | 2.0 | 265 | 0.24 | 5.0 | 3,797 | 690 | 18.2 | | 14 | 0.36 | 6.0 | 1.5 [0.91-2.62]^ns^ |
| 20-24 yo | 264,712 | 4,527 | 1.7 | 544 | 0.21 | 10.9 | 11,375 | 1,610 | 14.2 | | 55 | 0.48 | 12.6 | 2.4 [1.79-3.1]^****^ |
| 25-30 yo | 356,747 | 8,076 | 2.3 | 1,174 | 0.33 | 23.4 | 13,797 | 1,967 | 14.3 | | 94 | 0.68 | 22.5 | 2.1[1.68-2.55]^****^ |
| 30-34 yo | 349,159 | 10,072 | 2.9 | 1,866 | 0.53 | 38.7 | 10,888 | 1,707 | 15.7 | | 92 | 0.84 | 31.6 | 1.6[1.28-1.95]^****^ |
| 35-39 yo | 330,176 | 12,983 | 3.9 | 2,946 | 0.89 | 63.4 | 5,788 | 1,044 | 18.0 | | 82 | 1.41 | 56.2 | 1.6[1.28-1.97]^***^ |
| 40-44 yo | 303,662 | 16,070 | 5.3 | 4,479 | 1.48 | 99.5 | 1,536 | 279 | 18.2 | | 33 | 2.14 | 99.8 | 1.5[1.04-2.04]^*^ |
| **Total** | **1,715,472** | **53,982** | **3.1** | **11,274** | **0.66** | **38.5** | **47,181** | **7,297** | **15.5** | | **370 ^a^** | **0.78** | **23.1** | **1.2[1.08-1.32]^***^** |

^a^ The total deaths in the pregnant were 377, with 7 deaths outside the age groups depicted in this table (>44 yo).

^b^ For death rate estimations, the following number of pregnancies that produced live births, were used: 231,511; 436,140; 417,404; 291,140; 148,814; and 40,079, for 15-19, 20-24, 25-34, 35-39, 40-44 yo, respectively, according to INEGI:(<https://www.inegi.org.mx/contenidos/saladeprensa/boletines/2021/EstSociodemo/NamtosRegistrados2020.pdf>)

These numbers of live births were for 2020 and they are lower than in previous years, plus they do not include pregnancies that resulted in stillbirths or abortions. Thus, death rates in the pregnant may be slightly over estimated.

^c^ RR of death is the Relative Risk of death of the pregnant vs the non-pregnant in each age-group; 95% confidence intervals (CI) are shown.

ns, not significant P>0.05; * P<0.05; *** P< 0.001; **** P<0.0001; with Chi square with Yates correction.
