# Supplementary material for: Decrease in COVID-19 adverse outcomes in adults during the Delta and Omicron SARS-CoV-2 waves, after vaccination in Mexico
Source: Front Public Health. 2022 Sep 13;10:1010256. doi: 10.3389/fpubh.2022.1010256 (PMC9513220; doi:10.3389/fpubh.2022.1010256)
Supplement: Supplementary file 3 [file Data_Sheet_1.docx]

**Supplementary methods**. Clarification on Peak fitting with the program Magicplot.

Waves in the epidemic curves were detected and fitted as independent gaussian curves. That is, each peak is assumed to have an x-value matching the highest y-value (mid-peak), a specific width (distance from half of the maximal y), and these values are independent from other peaks. The end of one wave and the beginning of the next, were often assigned by the software, to dates where peaks overlap, so they were resolved by manually identifying inflexion points (minimal values between waves). Since the peaks were clearly defined and separated, any initial guess on the fit converged easily to the same result.
